# Knowledge of HIV and benefits of male medical circumcision amongst clients in an urban area

**DOI:** 10.4102/phcfm.v6i1.722

**Published:** 2014-12-11

**Authors:** Abidemi Faleye

**Affiliations:** 1 Department of Family Medicine, University of KwaZulu-Natal, Durban, South Africa

## Abstract

**Background:**

Male medical circumcision (MMC) has been shown to reduce the risk of HIV transmission in circumcised men by up to 60%. Following recommendations from the World Health Organization, South Africa adopted MMC as a preventative strategy against HIV in 2010 and set up circumcision camps across the country. Concerns have been raised about condom avoidance following MMC because of a mistaken belief about the benefits of MMC.

**Aim and setting:**

The aim of this study was to describe the profile and knowledge about HIV and circumcision amongst men presenting for MMC in an urban area in KwaZulu-Natal.

**Methods:**

This was a cross-sectional descriptive study of 394 clients over the age of 18 years who presented to two MMC sites in Durban between November 2012 and March 2013. A validated questionnaire was used to collect data.

**Results:**

The mean age of clients presenting for MMC was 28 years. Most clients were black, single, unemployed and sexually active. The majority presented for MMC because they believed that MMC would reduce their risk of acquiring HIV infection. Knowledge about HIV transmission was very good and 86.3% of clients were aware that risky sexual behaviour such as condom avoidance could reverse the benefits of MMC.

**Conclusion:**

The knowledge of HIV and benefits of MMC was very good amongst those presenting for MMC. However as MMC is primarily a preventative strategy, innovative methods to promote MMC prior to first sexual encounter need to be explored. Further research is needed to determine whether the benefits of MMC on the reduction of HIV transmission are sustained in routine practice.

## Abstrait

**La connaissance du VIH et les bienfaits de la circoncision médicale masculine chez les clients qui se présentent pour la circoncision dans les zones urbaines**.

**Contexte:**

Il a été démontré que la circoncision médicale masculine (MMC) réduit de 60% les risques de transmission du VIH chez les hommes circoncis. A la suite des recommandations de l'Organisation mondiale de la Santé, l'Afrique du Sud a adopté en 2010 la MMC comme stratégie de prévention contre le VIH et établi des camps de circoncision dans tout le pays. On a montré de l'inquiétude sur la non utilisation de préservatifs suite à la MMC due aux idées fausses sur les bienfaits de la MMC.

**Objectif et cadre:**

Cette étude avait pour but de décrire le profil et les connaissances sur le VIH et la circoncision chez les homes qui se présentent pour la MMC dans une zone urbaine du Kwa Zulu Natal.

**Méthodes:**

Ceci était une étude transversale descriptive de 394 clients de plus de 18 ans qui se sont présentés à deux services de la MMC à Durban de novembre 2012 à mars 2013. On s'est servi d'un questionnaire validé pour collecter les données.

**Résultats:**

L’âge moyen des clients se présentant pour la MMC était 28 ans. La plupart des clients étaient noirs, célibataires, chômeurs et actifs sexuellement. La majorité se présentaient pour la MMC parce qu'ils croyaient que la MMC réduirait le risque d’être infecté par le VIH. Ils étaient bien au courant de la transmission du VIH et 86.3% des clients savaient que les comportement sexuels risqués tel que la non utilisation des préservatifs pouvaient annuler les bienfaits de la MMC.

**Conclusion:**

Les hommes qui se présentaient pour la MMC avaient une bonne connaissance du VIH et des bienfaits de la MMC. Cependant, étant donné que la MMC est en premier lieu une stratégie de prévention, il faut trouver des méthodes innovatrices pour promouvoir la MMC avant la première relation sexuelle. Il faut faire des recherches supplémentaires pour déterminer si les bienfaits de la MMC sur la réduction de la transmission du VIH sont durables dans la pratique quotidienne.

## Introduction

Currently, 2.3 million new HIV infections occur each year and 35.3 million people worldwide are living with HIV.^1^ With such large numbers of people infected with HIV, prevention and treatment of HIV are an international priority. New prevention strategies such as male medical circumcision (MMC), use of vaginal microbicides, pre-exposure prophylaxis with anti-retroviral agents, herpes suppressive therapy, cervical barrier methods, HIV vaccines and early initiation of antiretroviral therapy are all being promoted as potential methods for the reduction of HIV transmission.^2^

Male medical circumcision (MMC) is the surgical removal of the prepuce from the glans of a flaccid penis. Epidemiological studies have shown a geographical correlation between male circumcision and HIV prevalence, with countries that traditionally practise male circumcision having lower rates of HIV prevalence than countries that do not.^3, 4^ Three randomised controlled trials done in South Africa (SA), Kenya and Uganda in 2005 and 2007 showed that MMC reduced HIV transmission in circumcised men by 60%, 53% and 51%, respectively.^5, 6, 7^ This reduction in HIV transmission results from the removal of the prepuce which contains a large number of Langerhans cells. These cells are immunological in nature and are rich in CD4 cells, making them a target for HIV entry and replication.^3^ In addition, MMC has been shown to protect circumcised men from ulcerative sexually-transmitted infections (STIs)^6, 7^ such as Herpes simplex virus type 2 (HSV-2), which predisposes one to HIV infection, as well as against human papilloma virus (HPV) infections.^8^ The reduced incidence of HPV infection in circumcised men has resulted in the reduction of penile carcinoma amongst circumcised men and indirectly protects women against cervical cancer.^9, 10, 11^ MMC has also been noted to lead to a reduced risk of prostate carcinoma, less inflammation and infection of the skin of the penis and better genital hygiene.^12^ A number of African studies have shown MMC to be an acceptable intervention with high levels of uptake.^3, 5^

In 2007, based on the evidence of these studies, an expert consultative forum of the WHO/UNAIDS (World Health Organization/Joint United Nations Programme on HIV/AIDS) recommended that MMC be introduced in countries with a high prevalence of HIV and a low prevalence of male circumcision as part of the comprehensive HIV prevention package offered in these countries.^2^ As SA meets these criteria, MMC was adapted in 2010 as part of the national strategy for reducing HIV transmission. Since then, a large number of circumcision camps have been set up across SA in order to provide MMC services to males willing to be circumcised, with a target of circumcising 80% of uncircumcised men over a period of five years.^13^

However, concerns have been raised regarding the adaptation of the WHO recommendation and taking this strategy to scale in the South African context. Ncayiyane argues that the scientific evidence for large-scale roll out of MMC is insufficient to justify the energy, money and resources which have been devoted to the roll out of circumcision services. He further posits that the potential increase in risky behaviour based on the belief that circumcision prevents transmission of HIV, termed risk compensation, could nullify any gains from circumcision.^14, 15^ This concern was supported by data from a 2003 study in the Westonaria district of SA which showed that 30% of circumcised men believed that they could safely have sex with many women without using condoms because they believed that circumcision prevented the transmission of HIV.^15^ However, in a study conducted in Johannesburg in 2010, Bridges showed that even though a section of the population found condom avoidance attractive post-circumcision, this risk was overstated in the 2003 study by Lagarde.^16^ In fact, a study done in Kenya in 2006, which examined attitude, beliefs and behaviour regarding HIV and MMC, showed that condom use went up in both circumcised and uncircumcised groups (i.e., control and experimental groups) following an intensive circumcision drive and this was linked to the counseling provided to men considering circumcision.^17^

Despite the concerns about condom avoidance in circumcised men, MMC is an important preventative intervention, as men who engage in risky sexual behaviour do not consistently use condoms and circumcision has been shown to lead to a significant reduction in the risk of HIV transmission in men.^18^ There is, however, a lack of information on the profile of men who access the MMC services in KwaZulu-Natal as well as their knowledge of HIV, their understanding of the benefits of MMC and their understanding of the importance of condom use post-MMC. This study aimed to address this gap and to make recommendations to the Department of Health regarding its circumcision campaign.

## Research methods and design

### Study design and setting

This was a cross-sectional descriptive study carried out at Wentworth and St Aidan's Hospitals in Durban, KwaZulu-Natal. Since March 2010, as part of the MMC roll-out programme, MatCH (Maternal Adolescent and Child Health) has been providing a free circumcision service to the population of Durban at Wentworth Hospital, St Aidan's Hospital and KwaMashu Polyclinic. Since the inception of the project, MatCH has run MMC educational programmes and recruitment drives in the townships around Durban and has provided free transport to the most convenient circumcision site. To date over 10 000 clients have been circumcised at these sites.

### Study population and sampling strategy

With up to 1000 clients presenting per month for MMC at Wentworth Hospital (WWH) in 2011, a sample of 400 clients representing 40% of those presenting in a month was considered sufficient for an observational descriptive study.^19^ Inclusion criteria were males 18 years or older presenting for MMC who agreed to participate in the study. There were no exclusion criteria. All clients who presented for MMC on the days that the research assistant was available were asked to participate in the study. No one who was approached refused to participate in the study. Prior to enrolment, patients were given an information sheet and informed consent was obtained by the research assistant. Data were collected prior to the MMC procedure using a questionnaire adapted from a validated questionnaire previously used by MatCH.^20^ Clients were asked to complete the questionnaire on their own and were assisted by the research assistant when necessary.

In October 2012, as a result of transport challenges associated with getting clients to WWH, the number of men presenting at the hospital for MMC dwindled significantly. As MatCH is also responsible for the MMC programme based at St Aidan's Hospital, where more than 50 clients per day were presenting for MMC, permission was requested from the ethics committee, the KwaZulu-Natal Department of Health and the hospital management to switch the study site from WWH and to gather data from St Aidan's from January 2013. Data were collected at WWH from November to December 2012 and from St Aidan's from January to March 2013.

### Data analysis

Data were captured onto a Microsoft® Excel spreadsheet and analysed using the Statistical Package for Social Sciences (SPSS) version 21 (IBM Corp., Armonk, NY, 2012).

### Ethical considerations

Approval for the study was given by the Biomedical Ethics Committee of the University of KwaZulu-Natal (reference number BE001/12), the hospital management and the KwaZulu-Natal Department of Health.

## Results

Three hundred and ninety-four clients completed the questionnaires. Seventy-eight (19.8%) were from WWH and 316 (80.2%) from St Aidan's hospital. The mean age of clients was 28 years (standard deviation 8.67). The demographic profile of the participants is shown in Table 1.

**TABLE 1:** Demographic profile of clients (*N* = 394).

Variable	Frequency	Percentage
**Race**		
Black people	356	90.4
White people	0	0.0
Indian	21	5.3
Mixed race	12	3.5
Missing	5	0.8
**Age**		
18–30	285	72.3
31–43	79	20.1
44–56	23	5.8
> 56	7	1.8
**Highest level of education**		
Never attended school	8	2.0
Attended primary school (Grade 1–Grade 7)	62	15.7
Attended high school (Grade 8–Grade 12)	145	36.8
Passed Matric	60	15.2
Post Matric training (either at college or university)	119	30.2
**Sexually active**		
Yes	392	99.5
No	2	0.5
**Marital status**		
Single	361	91.6
Married	30	7.6
Widowed	2	0.5
Divorced	0	0.0
Missing	1	0.3
**Job status**		
Unemployed	240	61.1
Employed	153	38.9
Missing	1	0.3
**Area/Address**		
Urban	154	39.9
Urban townships	210	53.3
Urban informal	19	4.8
Rural	11	3.0

The majority (*n* = 210, 53.3%) of the clients came from local urban townships, with a few presenting from other districts. The majority had attended high school; over 60% (*n* = 324) were unemployed, most were single and sexually active.

The majority of clients presented for MMC because it offered partial protection against HIV with only 30 (7.6%) having a circumcision because of social and/or religious reasons. Knowledge of transmission of HIV was excellent. Almost all of the clients had been counselled and tested for HIV and 85% (*n* = 337) were HIV negative.

Most of the clients had a good understanding of the effects of MMC and the risk of acquisition of HIV and understood that MMC is just part of the preventative package against HIV.

## Discussion

The majority of those who participated in this study were in the 18–30 year age group (72.3%), with an average age of 28 years as seen in Table 1. These figures may have been distorted by the exclusion for ethical reasons of clients less than 18 years of age (109 clients were aged less than 18 years). However, these figures are consistent with a study by Connolly in 2005 which reported a similar age distribution in a national survey of MMC.^21^

Almost all of those who participated in this study (99.5%) were sexually active. As MMC is intended to prevent HIV transmission it would be preferable to circumcise men prior to initiation of sexual relationships, especially in a society with a high prevalence of HIV. Pre-pubertal circumcision has been associated with the greatest reduction in the risk of HIV acquisition.^22^ Although the age of circumcision varies with race, tribe and religion,^14, 15^ local studies are needed in order to determine the ideal age for MMC and to assess the acceptability of neonatal circumcision as a strategy for the prevention of HIV in SA.

The majority of those who participated in this study cited partial protection against HIV and personal hygiene as their reasons for seeking circumcision, as depicted in Table 2. Surprisingly, social and/or religious and cultural reasons accounted for less than 8% of the participants. This was unexpected, as in 2007 King Zwelithini stated that circumcision was a well-established Zulu cultural practice and that all young men should be circumcised.^23^

**TABLE 2:** Knowledge of male medical circumcision, HIV infection and reason for seeking male medical circumcision amongst clients.

Variable	Frequency	Percentage
**Reasons for seeking MMC**		
Partial protection against HIV	338	85.8
Social and/or Religious reasons	30	7.6
Personal hygiene	329	83.5
Others	0	0.0
**Benefits and advantages of MMC**		
Improved sexual performance	8	2.0
Prevention of HIV transmission	315	79.9
Reduce risk of STIs	350	88.8
Cultural acceptability	17	4.3
**Knowledge about HIV**		
Yes	390	98.5
No	4	1.0
**Knowledge about HIV**		
Sexual intercourse	383	97.2
Blood transfusion	358	90.9
Mother to child	353	89.6
Sharing needles	356	90.0
Hugging and kissing	7	1.8
Others	2	0.5
**Previous VCT**		
Yes	388	98.5
No	4	1.0
Missing	2	0.5
**HIV status**		
Positive	56	13.2
Negative	337	85.0
Missing	1	0.3
**Duration of MMC healing**		
1 day	7	1.8
1 week	4	1.0
2 weeks	8	2.0
> 2 weeks	374	94.9
Missing	1	0.3
**Having sex whilst circumcision heals**		
Yes	11	2.8
No	381	96.7
Unsure	1	0.3
MMC, male medical circumcision; VCT, voluntary counselling and testing; HIV, Human-Immunodefiency Virus; STI, sexually-transmitted infections.		

In keeping with a Namibian study done in 2010,^24^ most clients had excellent knowledge of HIV, its transmission and benefits of circumcision. These men were aware that MMC provides only partial protection, that condoms are still needed to prevent HIV and that the benefits of MMC could be reversed if other protective lifestyle choices such as condom use are not adhered to (Tables 2 and 3). This is encouraging, as it suggests that HIV-related knowledge and the benefits of circumcision are reaching the target population. Recent findings have shown a significant drop in HIV prevalence in SA, which may suggest that not only is knowledge better but that HIV prevention messages are having an effect on sexual practices.^25^

**TABLE 3:** Effect of male medical circumcision and the risk of acquisition of HIV (*N* = 394).

Variable	Frequency	Percentage
**Effect of MMC on the risk of acquiring HIV**		
No effect	11	2.8
It increases the risk of acquiring HIV	6	1.5
It reduces the risk of acquiring HIV	375	95.2
Other	1	0.3
Missing	1	0.3
**Whether risky sexual behaviour can remove the benefits of male circumcision**		
Yes	340	86.3
No	35	8.9
Unsure	18	4.6
Missing	1	0.3
**Whether male circumcision protects women from being infected with HIV**		
Yes	34	8.7
No	347	94.9
Unsure	11	11.0
Missing	2	0.5

MMC, male medical mircumcision; HIV, Human Immunodefiency Virus.

MMC has been shown to reduce the risk of HIV transmission from men to women. Although most of those who participated in this study believed that circumcision does not protect women from being infected with HIV (Table 3), Alsallaq found that by reducing the pool of HIV-positive men, circumcision can indirectly protect women from HIV infection.^26^ This information appears to be lacking and could be used as another reason for men to consider circumcision.

Just over 13.2% of those who participated in this study were HIV-positive (Table 2). Initial Department of Health guidelines recommended MMC only for men who were HIV negative. However, the WHO currently recommends MMC for HIV-positive men for medical indications and because of the possible reduction in risk of transmitting HIV in those already infected with HIV.^2^ HIV-positive men also benefit from the reduced risk of ulcerative STIs which is associated with MMC.^7^ In 2012, the Department of Health updated its guidelines and currently recommends MMC in HIV-positive men if their CD4 is above 350 cells/mm^3^, as studies have shown good wound healing at such CD4 levels.^7^ Currently, most of the HIV treatment programmes across SA have more women than men on treatment.^27, 28^ This may be because women tend to access healthcare services more often than men and have more opportunities for testing than men (during antenatal care, when presenting at a clinic with a sick child, etc.).^27, 28^ There is a need to find innovative ways to encourage men to test for HIV and for those who are positive to participate in treatment programmes. With the ambitious target of reaching 80% of uncircumcised men, the MMC programme may present such an opportunity for testing and for channelling men who are HIV-positive into HIV treatment programmes.

### Limitations

This was a small study conducted at two sites in KwaZulu-Natal. Because of the convenient nature of the sampling method, the results of this study may not be representative of the study population and may not be generalisable to other settings. The exclusion of clients younger than 18 years of age was a limiting factor as their experience and knowledge may be different from older clients.

### Recommendations

More local studies are needed to gain a better understanding of the effect of MMC on sexual behaviour and to measure the impact of MMC on HIV transmission when it is incorporated into routine practice. There is also a need for research to be done amongst those under 18 years of age in order to determine their reasons for presenting for MMC and their knowledge of the benefits of MMC.

## Conclusion

This study found that the average man presenting for MMC at these MatCH sites is young, lives in an urban township and is black, single and sexually active. Those presenting for MMC have excellent knowledge of the effects of circumcision on the transmission of HIV, were aware that MMC provides only partial protection, that condoms are still needed to prevent HIV and that the benefits of MMC can be reversed if condom use is not adhered to. Although the concerns about risk compensation are legitimate, most of the men in this study understood that condom use is still necessary post-circumcision. Circumcision presents an opportunity to educate young men about HIV prevention and to channel those who are positive into HIV treatment programmes.
